# The Influence of Occupational Factors on Contact Dermatitis in Symptomatic Healthcare Workers: A Patch Test Study †

**DOI:** 10.3390/diseases13030077

**Published:** 2025-03-07

**Authors:** Cristiana Ferrari, Giuseppina Somma, Viola Giovinazzo, Margherita Iarossi, Michele Treglia, Margherita Pallocci, Luca Di Giampaolo, Andrea Magrini, Luca Coppeta

**Affiliations:** 1 Department of Biomedicine and Prevention, University of Rome Tor Vergata, 00133 Rome, Italy; giuseppina.somma@ptvonline.it (G.S.); viola.giovinazzo90@gmail.com (V.G.); margheritaiarossi@gmail.com (M.I.); michele.treglia@uniroma2.it (M.T.); margherita.pallocci@gmail.com (M.P.); andrea.magrini@uniroma2.it (A.M.); luca.coppeta@uniroma2.it (L.C.); 2 PhD. Program in Social, Occupational and Medico-Legal Sciences, University of Rome Tor Vergata, 00133 Rome, Italy; 3 Department of Occupational Medicine, University of Chieti “G. D’Annunzio”, 66013 Chieti, Italy; digiampaololuca@libero.it

**Keywords:** healthcare workers, patch test, skin sensitization, contact dermatitis

## Abstract

Healthcare workers (HCWs) are frequently exposed to a variety of chemical agents, which can result in the development of allergic or irritant contact dermatitis. The present study aimed to assess the prevalence of skin sensitization among HCWs who presented with symptoms of contact dermatitis, considering both occupational and non-occupational risk factors. The study population comprised 127 HCWs who attended routine occupational health surveillance at the Tor Vergata Teaching Hospital in Rome between November 2023 and May 2024. A structured dermatitis questionnaire and patch testing were administered to the participants. Demographic and lifestyle data, including information on occupation, night shift work, smoking habits, and body mass index (BMI), were collected. Patch test positivity was observed in 31.5% of participants, with the most common clinical presentation being erythematous-desquamative allergic contact dermatitis. A significantly higher likelihood of patch test positivity was observed among nurses (57.1%), particularly for nickel sensitization, compared to other occupational groups. A trend towards an association between night shift work and skin sensitization was observed, although this did not reach statistical significance. No significant associations were found for ages over 35 years, sex, or BMI. These findings highlight the elevated risk of contact sensitization among nurses, emphasizing the need for targeted interventions, including exposure reduction strategies and protective measures, to mitigate occupational skin hazards in healthcare settings.

## 1. Introduction

Contact dermatitis (CD) is a common inflammatory dermatologic condition resulting from exposure to allergens and contact irritants. It is the most common cause of occupational dermatitis. It can present symptoms similar to those of other dermatologic conditions, including atopic dermatitis, lichen planus, and angioedema [1,2]. It is an eczematous inflammatory dermatologic disease caused by chemicals or metal ions that exert toxic effects without inducing a T-cell response (contact irritants) or by reactive chemicals that induce innate and adaptive immune responses (contact allergens) [3,4,5,6,7,8,9].

The two major categories of contact dermatitis are allergic contact dermatitis and irritant contact dermatitis. Irritant contact dermatitis is a nonspecific cutaneous response to a direct chemical insult that results in the release of inflammatory mediators predominantly from epidermal cells. The condition is caused by repeated contact with solvents, detergents, or industrial materials that can damage the skin without actually activating the immunological response. Atopic contact dermatitis is a delayed-type hypersensitivity reaction (type 4) to exogenous contact antigens (allergens) that triggers an immune response in previously sensitized individuals. The immunological response is due to the interaction between cytokines and T cells. With photocontact, allergic dermatitis lesions are limited to sun-exposed areas. This is true even if the allergen is in contact with covered areas.

It is important to obtain a history of the patient’s occupation, hobbies, and any topical or oral medications taken to facilitate an accurate diagnosis of contact dermatitis.

Contact dermatitis (CD) represents one of the most prevalent work-related diseases in developed countries. Occupational contact dermatitis has been demonstrated to exert a detrimental effect on the quality of life of workers and their ability to perform their professional duties. CD is a significant problem in the healthcare sector, affecting an estimated 13% to 30% of workers in both irritant and allergic forms. Healthcare workers (HCWs) are considered to be at an elevated risk of developing contact dermatitis as a result of the repetitive handwashing with soaps and disinfectants and the prolonged use of gloves for extended periods during the course of their duties [10].

Those operators are often exposed to a variety of chemicals that can cause allergic or irritant contact dermatitis, aggravating existing skin conditions such as eczema and psoriasis. The prevalence of skin damage among frontline healthcare workers has been shown to be very high and to be significantly correlated with exposure time and, therefore, with longer work shifts [1].

Several studies have shown an increased incidence of skin conditions such as psoriasis and skin cancer in individuals with disturbed sleep patterns, including night shift workers. However, these studies do not address skin sensitization [11]. The coexistence of work-related dermatitis and underlying skin disease can lead to complex clinical presentations. Diagnosis of occupational contact dermatitis can therefore be challenging and may go undetected without targeted patch testing for specific occupational allergens [12]. Previous studies have shown a high rate of positive patch test results among healthcare workers, particularly for common sensitizing agents such as thiuram, quaternary ammonium compounds, formaldehyde, latex, and nickel [13]. Recent evidence suggests that rotating night shifts in hospitals may increase the risk of inflammatory skin diseases, possibly due to hormonal changes and circadian disruption. The dysregulation of the immune system caused by these factors, combined with occupational stress, may contribute to the development of inflammatory and allergic skin conditions. However, the potential combined effects of chemical sensitization and night shift work in healthcare workers have not been thoroughly investigated. The aim of this study was to investigate the prevalence of skin sensitization in healthcare workers with symptoms of contact dermatitis, taking into account their occupational risk factors and night shift status [14,15].

## 2. Materials and Methods

The study was conducted at the Tor Vergata Teaching Hospital, Rome, from November 2023 to May 2024. The selection of the study sample was conducted as part of the routine occupational health surveillance program. During the visits, individuals were asked if they had any skin symptoms suggestive of dermatitis, such as burning or itching. The presence or the absence of those symptoms were recorded in a specific dataset. Workers who exhibited suggestive symptoms were subsequently offered the opportunity to undergo patch testing to further investigate potential allergic or irritant causes of their dermatological condition. In total, 135 symptomatic HCWs were included in the study.

The patch test is considered the gold standard for the diagnosis of allergic contact dermatitis because it allows the precise identification of the causative agent. The chemicals included in the patch test kit are representative of a wide range of substances, including those found in metals (e.g., nickel), rubber, leather, formaldehyde, lanolin, perfumes, toiletries, hair dyes, drugs, pharmaceuticals, foods, beverages, preservatives, and other additives. The patch test is an invaluable diagnostic tool for identifying possible allergens that may cause a delayed-type hypersensitivity reaction. The test involves applying a dilute chemical solution to a small area of the patient’s back, which then induces a localized allergic reaction [16,17,18].

We used T.R.U.E. Test SmartPractice ^®^ test patches (SmartPractice Denmark ApS, Hillerød, Denmark) containing 40 haptens from the SIDAPA 2016 series (European Chemotechnique Diagnostic). Patches were applied to the subjects’ backs (interscapular region) and removed after 48 h, and final results are read 48–72 h later. Reactions are graded according to the guidelines of the International Contact Dermatitis Research Group guidelines: negative (−), irritant reaction (IR), equivocal/uncertain (+/−), weak positive (+), strong positive (++), and extreme reaction (+++).

Prior to the administration of patch testing, subjects were interviewed by an operator using a validate questionnaire [19] to ascertain pertinent anamnestic information, including details pertaining to their occupation, night shift status, smoking habits and body mass index (BMI). This information and those regarding skin symptoms suggestive of dermatitis were meticulously documented in a confidential dataset, accessible exclusively to personnel involved in the study.

The study population was classified regarding the night shift status in two groups: “night shift workers”, defined as individuals who worked two to seven 12 h night shifts per month, and “day shift workers”.

The assessment of workers’ exposure to chemical substances was conducted on the basis of the hospital’s risk assessment. The use of latex was found to be limited to a few types of gloves in the operating room, thus resulting in minimal exposure. Formaldehyde exposure was identified in pathology and surgical units, where it is used as a preservative and disinfectant. The evaluation further identified additional potential allergens, including glutaraldehyde, peracetic acid in sterilization areas, alcohol-based sanitizers and chlorhexidine in patient care units, quaternary ammonium disinfectants in cleaning services, and pharmaceutical compounds in the pharmacy. In addition, workers were exposed to thiurams, a class of chemical compounds which occur naturally and are commonly present in rubber gloves and medical equipment. Workers could also have been exposed to metals such as nickel, cobalt, and chromium, which are found in trace amounts in medical instruments and orthopedic implants. This comprehensive evaluation enabled us to account for relevant occupational exposures when interpreting patch test results.

Written informed consent was obtained from all participants. The study was formally endorsed by Ethics Committee of Policlinic of Rome Tor Vergata, with the protocol number assigned to the study being 41.18.

Data were analyzed using SPSS 25, and the results were assessed for frequency and relative risk using univariate and multivariate logistic regression.

## 3. Results

The initial population included 135 symptomatic workers. Of those, 8 were excluded from the study, 5 subjects because they refused to undergo patch test, and 3 because they were using corticosteroids or antihistamines at the time of testing. As shown in Table 1, the final study population comprised a total of 127 healthcare workers, of whom 28 were male and 99 were female. The mean age of the cohort was 35.57 ± 13.16 years. The majority of the study group were physicians (32, 25.2%) and nurses (28, 22%). Out of the subjects tested, 71% are non-smokers, 22% are smokers and 7% are ex-smokers. According to our analysis, smoking is not a statistically significant factor for possible influence in allergic contact dermatitis (see Table 1).

According to the data, 52% of the subjects examined had a positive family history of atopy and 75.6% had a positive personal history of atopy. Meanwhile, 39.4% had rhinoconjunctivitis and 13.4% had asthma. These data, together with the presence of atopic dermatitis in 56% of subjects, suggest a possible association between atopy and allergic contact dermatitis.

Patch test results were positive in 31.5% of the sample, with the most common clinical presentation being erythematous-desquamative allergic contact dermatitis (ACD) (55.9%). The most common positive substances were nickel sulfate hexahydrate 5% (18.9%), Mci/Mi (Kathon cg) 0.01% (6.3%) and potassium dichromate 0.5% (6.3%), formalin 1% (6.3%).

The association between patch test positivity (almost one allergen) and the main study variables is shown in Table 2.

The results suggest a strong statistical association between being a nurse and a positive patch test result (*p* < 0.05), whereas there is no evidence of a significant association between being older than 35 years and a positive patch test result. There may be a trend towards an association between night shift work and a positive patch test result. The analysis indicates that differences found among the positivity rate in subjects with normal BMI, or those part of overweight and obese groups, were not deemed to be statistically significant. This suggests that, although there is an increasing trend in the positivity rate with increasing BMI, there is no strong statistical evidence of a relationship between the BMI category and patch test result. Future studies with larger sample sizes and additional inflammatory or metabolic markers may provide further insight into whether there are indirect relationships between body composition and contact sensitization.

These results provide a comprehensive overview of the associations between positive patch test results and the variables of interest. Nursing occupation appears to be the most strongly associated factor with positive patch test results, while ages of over 35 years do not show a significant association. Night shift work may have some influence, but further studies are needed to confirm this trend. Considering the association between the positivity to patch test for specific allergens, nurses exhibited a higher and significant prevalence of nickel (44.8%), while no significant association was found for other substances.

We then build up a regression logistic model to evaluate the possible association between patch test positivity and the main independent variables collected in the study. Being a nurse was significantly associated with higher odds of a positive patch test (OR = 7.779, *p* = 0.000). Other factors (age over 35 years, gender, BMI and night shift work) did not show statistically significant associations with patch test results. Results are shown in Table 3.

## 4. Discussion

Skin sensitization, as documented by positive patch testing, was found in approximately one-third of workers with symptoms. This percentage indicates a higher rate of sensitization among nurses compared to the general population, highlighting a significant occupational health issue. In particular, nurses were significantly more likely to be sensitized to nickel sensitization, with nearly 60% of symptomatic nurses testing positive. This prevalence was much higher than previously reported; the increased susceptibility highlights the need for targeted interventions and protective measures to reduce exposure to potential allergens or irritants.

Nurses are regularly exposed to a variety of potential allergens and irritants as part of their routine duties. These allergens and irritants include disinfectants, soaps, latex gloves, hand sanitizers, and various medical instruments. A significant number of these materials, particularly those containing nickel, are known to cause allergic contact dermatitis (ACD). Published studies indicated that healthcare workers, particularly nurses, are at an elevated risk of developing occupational skin diseases due to their frequent contact with such substances [20,21]. The results of this study corroborate the above observations and suggest that the nursing profession is associated with specific risks that require the implementation of targeted preventive measures.

Nickel is one of the most common causes of allergic contact dermatitis, and healthcare workers are particularly susceptible due to their frequent use of nickel-containing instruments and devices. A meta-analysis by Jacob et al. (2018) [22,23] reported that the prevalence of nickel allergy is significantly higher in healthcare workers compared to the general population. Our study confirms this finding and indicates that nurses have a significantly higher likelihood of having a positive patch test for nickel. This correlation suggests that occupational nickel exposure in medical settings is a significant risk factor for the development of ACD.

Another critical factor contributing to the high incidence of positive patch test results among nurses is the nature of their work, which often involves frequent hand washing and the use of alcohol-based hand sanitizers. This “wet work” has been well documented as a risk factor for irritant contact dermatitis (ICD) and has the potential to compromise the skin barrier, thereby increasing the likelihood of sensitization to allergens such as nickel. Smith and Leggat (2020) [24] observed that healthcare workers are at an increased risk of developing hand dermatitis due to these frequent hygiene practices, which are critical for infection control but detrimental to skin health. This assertion is corroborated by a recent review, which elucidated that the high prevalence of hand eczema among HCWs is likely to be primarily attributable to the high volume of occupational wet work in healthcare, which incorporates frequent handwashing in conjunction with the wearing of occlusive gloves. Additionally, HCWs are exposed to allergens in the workplace, including rubber constituents and fragrances, which can lead to the onset of both irritant and allergic contact dermatitis of the hands [25].

Age, sex, BMI, and night shift work do not show statistically significant relationships with patch test positivity in this model.

The overall explanatory power of the model is relatively low (pseudo R-squared of 0.07707), indicating that there may be other important factors not included in this analysis that could better explain the variation in patch test results.

While the model as a whole is statistically significant, most of the individual variables (with the exception of nurse job) are not, suggesting that the relationship between these factors and patch test positivity may be more complex than this model captures.

Previous research has also linked night shift work to inflammatory skin conditions [14,15,19,26], suggesting a potential link between altered circadian rhythms and skin health. Occupational stress among healthcare workers may further exacerbate skin problems, highlighting the importance of addressing workplace stressors in the prevention of skin disease.

Our study has several limitations, including its cross-sectional design, non-random sample selection, and small sample size. Future research should focus on the implementation of environmental and hygiene measures to reduce skin irritation and sensitization. Education, training, and skin protection measures based on the existing literature and extensive use of barrier creams may help to reduce the risk of skin disorders among healthcare workers [27]. In addition, the use of nitrile gloves instead of latex gloves may have contributed to the low frequency of latex sensitization found in our sample, consistent with previous studies [28,29,30]. Further observational studies are needed to validate our findings and to guide future preventive measures.

Although this study provides valuable insights into the occupational health risks faced by nurses, further research is needed to fully elucidate these risks and to develop effective preventive strategies. In particular, longitudinal studies tracking the incidence of contact dermatitis over time and evaluating the effectiveness of different interventions would be beneficial. In addition, an investigation of the genetic predisposition to contact allergy and the influence of environmental factors could provide a more complete understanding of the prevention of occupational allergies among healthcare workers.

## 5. Conclusions

In conclusion, the logistic regression analysis clearly demonstrates the significant risk of positive patch test results, particularly for nickel, among nurses. This finding highlights the critical need for targeted interventions to protect nurses from occupational allergens and irritants. It is imperative that education and training, the use of alternative materials, the implementation of barrier creams, and environmental controls are used as essential strategies to mitigate these risks. By prioritizing the occupational health of nurses, healthcare facilities can ensure a safer and more sustainable working environment, which will ultimately improve the well-being of both healthcare workers and patients.

## Figures and Tables

**Table 1:** Main characteristics of the study population according to work task, sex, smoking habit, and family and personal history of atopy.

Characteristics	N (%)
Work task	127 (100)
medical doctors	32 (25.2)
medical students	33 (24.6)
nurses	28 (22)
other	25 (20)
dentist	4 (3)
radiologic technologist	2 (1.5)
laboratory technician	2 (1.5)
biologist	1 (0,7)
Sex	
Male	28 (22)
Female	99 (78)
Smoking habit	
Yes	28 (22)
No	90 (71)
Ex	9 (7)
BMI	
<25	85 (67)
25–30	31 (24)
>30	11 (9)
Family history of atopy	
Yes	66 (52)
No	61 (48)
Personal history of atopy	
Yes	96 (75.6))
No	31(24.4)
Atopic Dermatitis	
Yes	71 (56)
No	56(44)

**Table 2:** Prevalence of patch test positivity by sex, night shift, occupation (nurse or not), age, and BMI.

	Patch Test Results					
Category	Negative Result	%	Positive Result	%	Chi-Square	Significance (*p*-Value)
Sex						
Male	21	75.0%	7	25.0%	0.703	0.402
Female	66	66.7%	33	33.3%		
Night Shift						
No	63	74.1%	22	25.9%	3.754	0.053
Yes	24	57.1%	18	42.9%		
Nurse						
No	75	75.8%	24	24.2%	10.950	0.001
Yes	12	42.9%	16	57.1%		
Age ≥ 30						
No	40	67.8%	19	32.2%	0.025	0.873
Yes	47	69.1%	21	30.9%		
BMI						
<25	61	71.7%	24	28.2%	1.4034	0.4957
25–30	6	54.5%	5	45.4%		
>30	21	67.7%	10	32.2%		

*p* level of significance: *p* < 0.05.

**Table 3:** Logistic regression analysis of patch test positivity by age, sex, night shift, and nurse role.

Variable	Coefficient	Std Error	Z-Value	*p*-Value	2.5% CI	97.5% CI	OR	OR 2.5%	OR 97.5%
Intercept	−0.372	0.757	−0.492	0.623	−1.857	1.112	0.689	0.156	3.041
Age	−0.028	0.02	−1.382	0.167	−0.067	0.012	0.973	0.935	1.012
BMI	−0.004	0.004	−0.994	0.320	−0.012	0.004	0.996	0.988	1.004
Sex	0.214	0.525	0.407	0.684	−0.815	1.242	1.238	0.443	3.463
Nurse	2.051	0.55	3.732	0.000	0.974	3.129	7.779	2.649	22.845

*p* level of significance: *p* < 0.05.

## Data Availability

The original contributions presented in this study are included in the article. Further inquiries can be directed to the corresponding author.
